# The effectiveness of intratissue percutaneous electrolysis for the treatment of tendinopathy: a systematic review

**DOI:** 10.17159/2078-516X/2022/v34i1a12754

**Published:** 2022-01-01

**Authors:** D Augustyn, A Paez

**Affiliations:** 1 College of Professional Studies, Northeastern University, 360 Huntington Ave, Boston, MA 02115, USA; 2 Nuffield Department for Primary Care Health Sciences, University of Oxford, Radcliffe Primary Care Building, Woodstock Rd, Oxford OX2 6GG, UK

**Keywords:** tendinosis, intratissue percutaneous electrolysis, percutaneous electrolysis

## Abstract

**Background:**

Tendinopathy is highly prevalent in the general public and common in athletes. It makes up nearly 50% of all sport injuries. A number of treatment techniques with varying evidence of effectiveness are currently available. Intratissue percutaneous electrolysis (EPI) is one such modality, however little consensus exists for EPI’s efficacy or the most effective treatment parameters.

**Objective:**

To review and appraise available evidence for Intratissue Percutaneous Electrolysis (EPI) in the treatment of tendinopathy, examining the effectiveness of EPI in conjunction with other modalities and identifying the strengths and limitations of the evidence base for EPI in order to make evidence-based recommendation for future studies of EPI.

**Methods:**

PubMed, Embase and Scopus were searched with keywords related to EPI and tendinopathy. Grey literature searches were conducted with Embase, OpenGrey, and ProQuest. Extensive citation searching was undertaken. Randomised controlled trials (RCTs), uncontrolled and observational studies of the application of EPI in patients aged 18–65 years with Magnetic Resonance Imaging (MRI) or clinical Ultrasonography (US) confirmed diagnosis of tendinopathy were eligible.

**Results:**

Eleven studies met inclusion criteria: six randomised control trials (RCTs) and five uncontrolled studies. Clinical trials of EPI as an adjunct modality with physical therapy reporting greater decreased pain and return to function than treatment with physical therapy alone. The evidence for EPI is limited and influenced by small sample sizes, varying treatment protocols, clinical heterogeneity and high risk of bias.

**Conclusion:**

It is currently not possible to conclude that EPI is an effective modality for the treatment of tendinopathy. RCTs with clearly described EPI treatment protocols, larger sample sizes and intervention reporting sufficient to support reproducibility are needed to determine the effectiveness of EPI for the treatment of tendinopathy.

Tendinopathy is a chronic overuse injury characterised by tendon degeneration, resulting in pain and decreased activity level. Tendinopathy is highly prevalent in the general public and common in athletes, representing up to 50% of all sports injuries.^[1]^ The term ‘tendinopathy’ is generally used to describe tendon disorders that include acute tendonitis, as well as chronic tendinosis.

From acute tendinitis to chronic tendinosis, it is important to recognise that each stage of the tendinopathy process involves specific histological changes, and that each stage of the disease has the potential to respond differently to various treatment modalities.^[2]^ Tendinitis refers to tendon inflammation and results from microtears that happen when the musculotendinous unit is acutely overloaded with a tensile force that is too heavy for it and/or applied too suddenly. Tendinitis lasts up to three weeks.^[2]^ Tendinosis is characterised by a chronic evolution of the degenerative process, including the formation of fibrotic tissue, degradation of myxoid substance, and decreased capillary blood flow, resulting in stagnation of the inflammatory cells (neutrophils and macrophages) required for phagocytosis. ^[2]^

Many treatment techniques, with varying evidence of effectiveness, are currently used to treat tendinopathy including rest, physiotherapy, eccentric exercise,^[3]^ extracorporeal shockwave therapy,^[3]^ non-steroidal anti-inflammatory drugs, corticosteroids, and platelet-rich plasma injections.^[4]^ Despite this, up to 29% of patients with tendon injuries develop chronic tendinopathy and require surgical management.^[5]^ Patients with decreased functional status compared to preinjury level and increased pain before treatment may also be less likely to return to a pre-tendinopathy functional level.^[5]^

Intratissue percutaneous electrolysis (EPI) is one modality for the treatment of chronic tendinopathy.^[6]^ Various synonymous terms for EPI are found in the literature, including ultrasound-guided galvanic electrolysis technique (USGET),^[7]^ percutaneous micro electrolysis (MEPV),^[8]^ percutaneous needle electrolysis (PNE),^[9]^ and ultrasound-guided percutaneous electrolysis.^[10]^ Intratissue percutaneous electrolysis is an ultrasound-guided physiotherapeutic technique in which electrical stimulation is applied to the injured tendon via an acupuncture needle to produce localised inflammation at the treatment area and stimulate tendon healing .^[6]^ Intratissue percutaneous electrolysis utilises a combination of mechanical (needle) and electrical (galvanic current) stimulation to provide controlled micro-trauma and non-thermal electrochemical ablation directly to the area of the degenerated tendon. This leads to the production of sodium hydroxide molecules, altered pH and increased oxygen in the treatment site, enabling cellular phagocytosis, and activating tendon repair.^[6,7]^

Little consensus exists for EPI’s efficacy or the most effective treatment protocol. To the best of our knowledge, no systematic review of EPI treatment had been published at the time this systematic review was undertaken. A systematic review of EPI is needed to critically appraise the evidence for its effectiveness and provide recommendations for clinicians and future research on its use in the treatment of tendinopathy. The primary objective of this review is to examine, categorise and critically appraise available evidence for EPI in the treatment of tendinopathy to determine if EPI is a safe and effective treatment for tendinopathy, identifying the strengths and limitations of the current body of evidence and making evidence-based recommendations for future research.

## Methods

This systematic review was undertaken with the guidance of the Cochrane Handbook for Systematic Reviews of Interventions, the Centers for Review and Dissemination (CRD), the University of York Guide, and reported according to the recommendations of the Preferred Reporting Items for Systematic Reviews and Meta-Analyses (PRISMA) statement.^[11]^ The review protocol was registered on PROSPERO: http://www.crd.york.ac.uk/PROSPERO/display_record.php?ID=CRD42018118345

### Information sources

Keywords and medical subject headings (MeSH) related to tendinopathy, tendinosis, intratissue percutaneous electrolysis, and percutaneous electrolysis were used in searches across multiple search engines and databases. No publication date or language limits were imposed.

Searches were undertaken on PubMed, Cinahl, Embase, Scopus, and the Cochrane library. The PROSPERO was searched for ongoing or recently completed systematic reviews addressing the review questions. To include EPUBS available ahead of the full publication, PubMed and Embase were searched again with the same methods, limiting results to articles added within the previous 90 days. Extensive searches in Embase, OpenGrey, Scopus, and ProQuest-Digital Dissertations were conducted for grey literature, including academic and conference papers. To ensure literature saturation, the reference lists of relevant papers were also searched, and citation searches were run on Scopus and PubMed.

### Inclusion criteria

Randomised controlled trials, uncontrolled and observational studies of the application of EPI in patients aged 18–65 years with clinical Ultrasonography (US) or Magnetic Resonance Imaging (MRI) confirming diagnosis of tendinopathy were eligible. Patients included in these studies had to have tendinopathy symptoms present for more than three weeks, the time frame where post-inflammatory (tendinitis) degeneration is present.^[1]^

### Exclusion criteria

Single subject case studies, case series, and animal studies were excluded. Studies with patients with bilateral symptoms, or who had undergone prior surgery for tendinopathy or received corticosteroid injections or used non-steroidal anti-inflammatory drugs during treatment were excluded. Studies of EPI not used in tendons were ineligible.

### Study selection

Search results were organised and collated into an electronic bibliographic database using RefWorks Citation Manager. Duplicate articles were identified and removed before proceeding with the screening process. Eligibility assessment was performed independently by two reviewers. Papers were initially screened against the review’s inclusion and exclusion criteria by title and abstract, then by full-text if eligible. A full-text review was also performed when eligibility was unclear by title or abstract. When necessary, study authors were contacted for additional information to resolve questions about their study’s eligibility.

### Data synthesis

A custom Excel spreadsheet was developed to collect data about and summarise included articles and to quantify extracted information. Information related to study design, sample characteristics, EPI methods, and outcome measures was extracted into the spreadsheet. Simple, descriptive statistics were used to quantify the results of the literature searches, screening, and systematic reviews.

### Risk of bias and quality assessment

To assess the methodological quality of the included articles, two different checklists were used. For the articles related to descriptive epidemiology and aetiology, the Quality in Prognosis Studies (QUIPS) tool was used (Appendix 1). The Cochrane Collaboration’s tool was used for the articles related to prevention (Appendix 1). For both the QUIPS and the Cochrane Collaboration’s tool, six potential bias domains were assessed with a high, moderate or low risk of bias. For assessments using the QUIPS tool, a study was considered to have a low risk of bias rated as low or moderate in all six domains, with at least four domains being rated as low.^[12]^ If two or more domains were scored as high, the study was rated as having a high risk of bias.^[12]^ Studies that were in between were scored as having a moderate risk of bias.^[13]^ For assessments using the Cochrane Collaboration tool, a study was assessed with a low risk of bias when all items were assessed as low.^[13]^ When at least one item was assessed as moderate, the article received a score with a moderate risk of bias. A high risk of bias was rated when at least one item was assessed as high.^[13]^ The checklists were assessed and cross-checked by two researchers (DA and AP). If a difference of opinion arose concerning the scoring of an item, a consensus was reached.

The Cochrane Risk of Bias (ROB) tool^[12]^ was used for randomised controlled trials. The ROB is widely available, and its reliability and validity have been shown previously.^[13]^ The Cochrane ROB was chosen based on the authors’ experience and prior training with the ROB and following the recommendations of the Cochrane Collaboration Guide to Systematic Reviews. The ROB focuses on different aspects of trial design, conduct, and reporting across seven domains of bias (Table 6). A series of signalling questions within each domain elicits information about the features of a trial relevant to the risk of bias. An algorithm is used to generate a judgement about the risk of bias for each domain based on the answers to the signalling questions. Judgements can be classified as ‘low’ risk of bias, ‘some concerns’ or ‘high’ risk of bias. ^[12,13]^

The framework for assessing the internal validity of prognostic studies developed by Altman (Table 1), posing key questions about study quality and the trustworthiness of studies’ results was used to assess the quality of all other study types.^[14]^

## Results

**Fig. 1:**
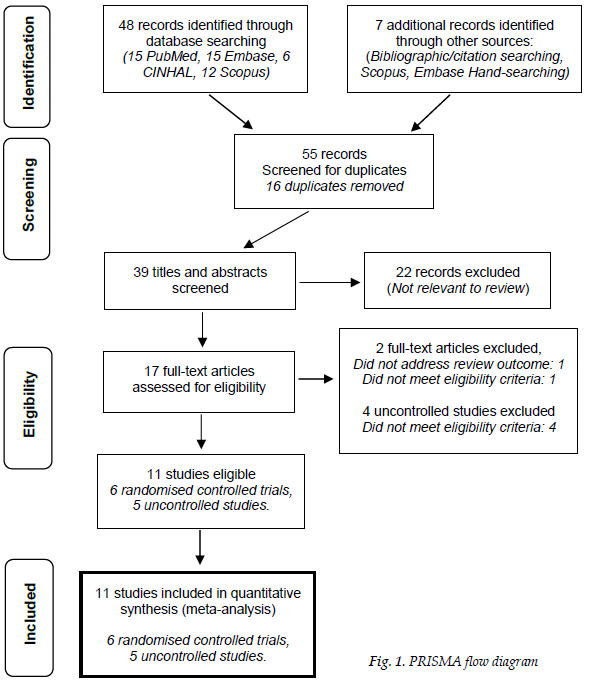
PRISMA flow diagram

Fifty-five papers were found: 48 in pre-specified search engines and seven from citation and bibliographic searching (Figure 1). After duplicates and ineligible papers were removed, 17 papers were screened and assessed against inclusion and exclusion criteria. Eleven papers met full eligibility criteria, including six randomised controlled trials (RCT) and five uncontrolled studies (Table 2). The results, including effect estimates and 95% confidence intervals of RCTs, are presented in Table 3, and the results of the uncontrolled studies in Table 4. Studies’ EPI treatment parameters, including intensity, duration, and the number of sessions are presented in Table 5.

A variety of outcome measures for patient-reported pain levels were used across eligible studies, including the Numerical Pain Rating Scale (NPRS),^[10,11]^ the Numerical Rating Scale (NRS),^[15]^ the Visual Analogue Scale (VAS),^[8,9]^ and the Shoulder Pain and Disability Index (SPADI).^[16]^ Each of these scales is used to rate pain from zero (no pain) to 10 (worst pain).

### Randomised controlled trials (RCTs)

Of the six eligible RCTs, three investigated EPI treatment of shoulder tendons,^[10,16,17]^ and one each for the knee,^[7]^ ankle,^[8]^ and thigh tendons.^[15]^ Primary outcome measures used by two shoulder tendon studies was the VAS,^[10,17]^ the third used the DASH questionnaire.^[16]^ The knee tendon study utilised the VISA-P scale, the ankle study the VISA-A scale, and the thigh study the VAS as primary outcome measures. Three of the RCTs used a treatment intensity between two and six milliampere (mA) with a maximum of five seconds active EPI up to three times per session;^[7,15,17]^ however, Abat et al.^[7]^ applied EPI to the area until complete debridement without time specification. The three remaining RCTs used intensities between 100 and 450 microamperes (μA) with a treatment duration up to 90 seconds.^[8,10,16]^ The characteristics of each study, as well as primary outcome measures, are listed in Table 2. Results are presented in Table 3, and the treatment protocols in Table 5. Quality assessment results are presented in Table 6.

### Uncontrolled studies

Five prospective, uncontrolled studies were eligible for inclusion.^[6,9,18,19,20]^ Three investigated EPI treatment in patellar tendon tendinopathy using the VISA-P scale as the primary outcome measure.^[6,18,19]^ One study had a 10-year follow up (88% completion rate),^[6]^ one a two year follow up^[19]^ and one a six week follow up (both 100% completion rate).^[18]^ Two studies investigated lateral epicondylitis of the elbow and used the VAS and the DASH questionnaire as primary outcome measures,^[9,20]^ with 6 week follow-up periods (100% completion rate). All uncontrolled studies utilized a treatment intensity of three to six milli ampere, three studies utilized a duration of three to five seconds performed three times,^[9,18,20]^ while two studies applied EPI until the area was debrided.^[6,19]^ The Characteristics of each study as well as primary outcome measures are listed in Table 2. Results are presented in Table 4, and treatment protocols are listed in Table 5. Quality assessment was performed using Altman criteria in Table 1.

### Quality assessment

Quality assessment of RCTs with the Cochrane ROB (Table 6) and other studies with Altman’s criteria (Table 1*)* resulted in a high risk of bias in all of the RCTs and uncontrolled studies eligible for inclusion in this systematic review. The greatest risk of bias among RCTs was found in the blinding of outcome assessment and selective reporting. Among uncontrolled studies, risk of bias was found in all studies, ranging low^[9]^ to high risk.^[6,18,19,20]^

## Discussion

Intratissue percutaneous electrolysis (EPI) is an innovative treatment technique for a musculoskeletal condition affecting a large portion of the general population. Results from this systematic review indicate that EPI shows promise as an adjunct modality in the treatment of tendinopathy when combined with exercise or manual therapy, but insufficient quality evidence is currently available to determine whether EPI is an effective treatment for tendinopathy. Relatively small sample sizes, heterogenic EPI treatment parameters and comparator interventions, and a high risk of bias found across available studies makes it difficult to reach definitive conclusions about EPI’s effectiveness.

The scope and quality of evidence for EPI are limited. No RCTs were found investigating EPI in comparison to a placebo adjunct modality or placebo intervention. Thus it is not currently possible to differentiate between the placebo benefit of a modality added to other interventions, such as exercise or manual therapy, and the true effects of EPI. Additionally, almost half (five of 11) of eligible studies were uncontrolled clinical trials, which can offer preliminary evidence of safety and indicate if there may be a clinical effect worth investigating further but cannot offer evidence of efficacy.^[21]^ The demonstration of a treatment’s efficacy requires a comparison of the response in the treated group with that of a control group receiving a placebo or another active treatment.^[21]^ Patients reported a return to function and reduced pain in four uncontrolled studies of EPI together with eccentric exercises, though it is impossible to attribute this to EPI in these studies as there were no comparison groups.^[6,9,17,18]^ There were no adverse events reported with EPI treatment in the five uncontrolled studies during treatment or at follow-up; however, indicating that EPI may be a safe procedure in the treatment of tendinopathy.

The high risk of bias found across all eligible studies may have been influenced by heterogenic treatment dosages, participant characteristics and outcome measures, and incomplete reporting of intervention details. A variety of treatment dosages were identified in the review, with EPI treatment intensity variously reported in milliamperes (mA) and microamperes (μA). The most consistent EPI treatment dosage reported was four-six mA for three sets of three seconds, though other dosages included 350 μA or two mA.^[9,18,20]^ Treatment duration lasting between four and 90 seconds in two studies,^[10,16]^ and three other studies describe treatment until the area was “fully debrided,” but how that was characterised or measured was not specified.^[6,7,19]^

Intratissue percutaneous electrolysis treatment was investigated as an added modality with various interventions among eligible studies, ranging from EPI and eccentric exercise,^[8,10]^ active physical therapy,^[15]^ manual therapy,^[20]^ electrophysiotherapeutic treatment,^[7]^ and general exercises,^[16]^ though these were often not reported sufficiently for reproducibility. Control interventions, or cointerventions within the EPI group, were not well described across eligible studies. Cointerventions in both the EPI and control groups may have influenced treatment outcomes separately from EPI tendon treatment. For example, Moreno^[16]^ investigated stand-alone EPI treatment of adductor tendons in a study of four groups of 10 patients each but does not report if any other treatment was received in addition to EPI, and the treatment protocol was not described in detail. Greater decreased pain was reported by the EPI group than the control groups in both the tendon and a muscular trigger point, though the study investigated the effect of EPI on tendinopathy, rather than trigger points.

Differences in the reporting of results and outcome measures used also make an assessment of EPI effectiveness difficult. Three RCTs presented results supported only by p-values, without reporting confidence intervals.^[8,17]^ One RCT presented results based on VISA-P scores categorically as greater or lesser than 90 at follow-up,^[7]^ greatly reducing reported details of EPI treatment effects. One RCT utilised a goniometric range of motion values at the shoulder as outcome measures without describing the measuring procedure, its validation, or reliability, raising the risk bias in the study,^[17]^ and only three studies assessed EPI in the same joint, further limiting the generalisability of the available evidence.^[10,16,17]^ An uncontrolled study comparing EPI to surgery focused outcomes on estimated cost, making direct comparison of treatment effects among both interventions difficult.^[20]^ Notably, none of the current studies investigated EPI for the treatment of tendonitis in the elbow, despite its high prevalence in the general population.

All of these factors may also have influenced the inconsistent findings reported among eligible studies. One RCT found improved outcomes in both the EPI and control groups,^[7]^ while some studies only reported improved outcomes in the EPI groups.^[8,12,15,17]^ One study found small improvements in pain but not in function for the EPI group,^[17]^ and one study found no differences between the groups.^[16]^

EPI is a complex intervention, with a number of independent and interdependent factors potentially influencing the effects of EPI treatment. These may include not only EPI dosage but also interventionist experience or a learning curve with EPI, patient and practitioners’ perception of equipoise and characteristics. Differences in practitioners’ skill with EPI and their inter- and intrapractitioner reliability, may also influence the outcome of EPI treatment and need to be established and further explored in future research. Future studies of EPI may benefit from the guidance of frameworks such as IDEAL-Physio or IDEAL,^[21]^ which is an established framework for guiding evidence-gathering in complex, practitioner-based interventions like EPI. The IDEAL framework prioritises transparent reporting of intervention details and delivery, consideration of pratitioner learning curves or skill with the intervention, standardisation of patient outcomes, and the selection of appropriate study designs for the level of development of innovative complex interventions like EPI.^[21]^

## Conclusion

Clinical trials investigating EPI as an adjunct modality with physical therapy report greater decreased pain and return to function than treatment with physical therapy alone, but the evidence for EPI treatment is limited and influenced by clinical heterogeneity, high risk of bias and small sample sizes. Therefore, it is not possible to definitely conclude that EPI is an effective modality for the treatment of tendinopathy. Randomised controlled studies with clearly defined and described protocols for EPI treatment, larger sample sizes, better defined control interventions, and reporting sufficient to support reproducibility are needed to determine the effectiveness of EPI as an adjunct modality in the treatment of tendinopathy.

## Supplementary Information



## Figures and Tables

**Table 1:** Quality assessment for assessing internal validity of studies, adapted from Altman, 2001 ^[13]^

Study feature	Qualities sought*
**Sample of patients**	Were the Inclusion criteria defined? Were sample characteristics described? Was sample selection explained? Did recruitment take place at the common stage or defined period? Was the sample representative of the population it is drawn from?
**Follow-up of patients**	Was follow-up long enough for the clinical outcomes (5 years)? Was follow-up complete (80%)?
**Outcome assessment**	Was it objective or independently adjudicated? Was the method standardized with objective tools? Was the outcome fully defined?

* Low risk of bias: Meets all or most of the criteria for a study feature; or

High risk of bias: Meets less than half of the criteria for a study feature

**Table 2:** Characteristics of studies included

Study and type	Joint	Population size	Intervention and exposure	Outcome measure
**Arias-Buria et al., 2015** ^[10]^ **Randomised controlled trial**	Shoulder	EPI group (n=17)	1 session EPI per week for 4 weeks applied to supraspinatus tendon and Eccentric exercise	VAS* DASH †
		Exercise group (n=19)	Similar Eccentric exercise as EPI group	

**Valtiera et al., 2018** ^[16]^ **Randomised controlled trial**	Shoulder	Control group (n=25)	Manual therapy for 5 sessions and exercise x2 per day for 5 weeks	DASH*, SPADI †, GROC†
		EPI group (n=25)	Manual therapy and exercise as control, and EPI with every manual therapy session	

**Moreno MD, 2015** ^[17]^ **Randomised controlled trial**	Shoulder	Control group (n=10)	Continue daily activities	VAS*
		EPI muscle group 1 (n=10)	EPI applied to trigger points in the muscle belly	
		EPI tendon group (n=10)	EPI applied only to the tendon of the infraspinatus	
		EPI muscle and tendon group (n=10)	EPI applied in trigger points and tendon	

**Abat et al., 2016** ^[7]^ **Randomised controlled trial**	Knee	Control group (n=30)	50 minutes of EPT 3x/week for 8 weeks and Eccentric exercise	VISA-P *
		EPI group (n=30)	1 EPI session every 2 weeks and similar Eccentric exercise as a control	

**Ronzio et al., 2017** ^[8]^ **Randomised controlled trial**	Ankle	Control group (n=10)	Friction massage of Achilles and Eccentric exercise of the plantar flexors	VISA-A* VAS*
		EPI group (n=10)	Same treatment as group 1 as well as EPI	

**Moreno et al., 2017** ^[15]^ **Randomised controlled trial**	Thigh	EPI group (n=11)	EPI and APT program	VAS* PSFS†
		Control group (n=13)	APT program	

**Valera - Garrido et al., 2010** ^[18]^ **Uncontrolled study**	Knee	32 patients observational study	One session EPI as well as eccentric training program per week for 4–6 weeks	VISA-P*

**Valera - Garrido et al., 2014** ^[9]^ **Uncontrolled study**	Elbow	36 patients	One session EPI per week 4–6 weeks as well as a home eccentric exercise program and stretching	DASH* Ultrasound image changes†

**Abat et al., 2014** ^[19]^ **Uncontrolled study**	Knee	33 patients studied prospectively	1 session of EPI treatment (average of 4,5 sessions per patient) per week and 2 Eccentric exercise sessions per week	VISA-P*

**Abat et al., 2015** ^[6]^ **Uncontrolled study**	Knee	40 patients studied prospectively	EPI treatment up to a maximum of 10 sessions or until symptom-free in conjunction with Eccentric exercise program	VISA-P*

**Munoz et al., 2012** ^[20]^ **Uncontrolled study**	Elbow	36 patients – cost effectiveness study	One session EPI, Eccentric exercise program and stretching per week for 4–5 weeks	VAS* DASH*

* indicates primary outcome measure;

† indicates secondary outcome measure.

EPI, intratissue percutaneous electrolysis; VAS, visual analogue scale; DASH, disabilities of the arm, shoulder and hand; SPADI, shoulder pain and disability index; GROC, global rating of change; EPT, electrophysiotherapeutic treatment; VISA-P, Victorian Institute of Sport assessment – Patella; VISA-A, Victorian Institute of Sport assessment – Achilles; APT, active physical therapy; PSFS, patient-specific functional scale.

**Table 3:** Results of randomised controlled trials

Study	Group	Population characteristics*	Measure instrument	Effect estimate and 95%CI†
**Arias-Buria et al., 2015** ^[10]^	EPI	4/13, 58 ± 7	VAS	−5.6(−6.4, −4.7) ‡
			DASH	−46.3(−52.2, −40,5) ‡
	Exercise	5/14, 57 ± 6	VAS	−3.7(−4.6, −2.9)
			DASH	−36.8(−42.2, −31.4)

**Valtiera et al., 2018** ^[16]^	Control	12/13, 55.3 ± 11.1	DASH	4.1 ± 3.4 (3.1, 5.1)
			VAS	27.6 ± 17.1 (22.7, 32.5)
			SPADI	−9.9 (−20, −0.3) §
	EPI	11/14, 54.9 ± 13.7	DASH	1.5 ± 1.8 (2.3, 3.3)
			VAS	10.1 ± 6.5 (4.7, 15.5)
			SPADI	

**Moreno MD., 2015** ^[17]^	Control	--,39.6 ± 3.69	VAS	-- (−4.9, 1.9)
	EPI muscle	--, 40.4 ± 3.20	VAS	-- (1.7, 24.3)
	EPI tendon	--, 39.9 ± 4.15	VAS	-- (54.6, 66.4)
	EPI muscle and tendon	--, 39.8 ± 4.66	VAS	-- (66.9, 74.1)

**Abat et al., 2016** ^[7]^	Control	24/8, 30.9 ± 5.9	VISA-P <90 (n=19)	61.9 ± 13.7 (55.3–68.5)
			VISA-P >90 (n=11)	95.2 ± 2.5 (93.5–96.9)
	EPI	27/5, 31.2 ± 6.5	VISA-P <90 (n=8)	63.3 ± 14.3 (51.3–75.2)
			VISA-P >90 (n=22)	97.1 ± 1.7 (96.3–97.8)

**Ronzio et al., 2017** ^[8]^	Physiotherapy	--	VISA-A, VAS	No values provided
	EPI	--		

**Moreno et al., 2017** ^[15]^	EPI	11/0, 26.9 ± 4.5	VAS on palpation VAS on contraction	1.1 ± 0.9 (--)
				0.5 ± 0.7 (--)
	Control	13/0, 25.2 ± 4.9	PSFS	95.4 ± 4.1 (--)
			VAS on palpation	2.0 ± 1.5 (--)
			VAS on contraction	1.6 ± 1.3 (--)
			PSFS	89.9 ± 6.8

* Population characteristics values are: male/female, mean age in years ± SD;

† Values are mean ±SD (95% confidence interval).

‡ denotes change from baseline value.

§ indicates difference between groups; -- indicates no values provided.

EPI, intratissue percutaneous electrolysis; VAS, visual analogue scale; DASH, disabilities of the arm, shoulder and hand; SPADI, shoulder pain and disability index; VISA-P, Victorian Institute of Sport assessment – Patella; VISA-A, Victorian Institute of Sport assessment – Achilles; PSFS, patient-specific functional scale.

**Table 4:** Results of uncontrolled studies

Study	Group	Population characteristics*	Measure instrument	Effect estimates (mean ± SD)	

				Baseline	Final follow-up
**Valera-Garrido et al., 2010** ^[18]^	Group 1 (n=13)	19/13, 35 ± 8.0	VISA-P <50	33 ± 8	69 ± 7
	Group 2 (n=19)		VISA-P >50	66 ± 7	88 ± 7 (6 weeks, n=32)

**Valera-Garrido et al., 2014** ^[9]^	n=36	19/17, 38 ± 6.4	VAS	60.2 ± 8.0	6.0 ± 12.0
			DASH	63.6 ± 9	13.6 ± 4.1 (6 weeks)

**Abat et al., 2014** ^[19]^	n=33	29/4, 25.3 (16–53)			
	Group 1†		VISA-P <50	31.5 ± 10.9	81.8 ± 14.5
	Group 2†		VISA-P >50	68.7 ± 10.3	89.4 ± 7.6 (2 years, n=33)

**Abat et al., 2015** ^[6]^	Group 1 (n=21)	17/4, 26 ± 8.49	VISA-P <50		88.8 ± 10.1
	Group 2 (n=19)	18/1, 25.7 ± 8.12	VISA-P >50		96.0 ± 4.3 (10 years, n=34)

**Munoz et al., 2012[20]**	n=36	19/17, 38 ± 6.4	VAS	--	--
			DASH	37.4 (--)	63.4 ± 9

* Population characteristics values are: male/female, mean age in years ± SD;

† n values per group unknown; -- indicates no values provided.

VAS, visual analogue scale; DASH, disabilities of the arm, shoulder and hand; VISA-P, Victorian Institute of Sport assessment – Patella; VISA-A, Victorian Institute of Sport assessment – Achilles.

**Table 5:** Treatment protocol used for randomized controlled trials and uncontrolled studies

Study	Joint	Needle	Intensity	Duration
**Arias-Buria et al., 2015** ^[10]^ **Randomised controlled trial**	Shoulder	0.3×25mm,	350μA - modified according to patient	90 seconds
**Valtiera et al., 2018** ^[15]^ **Randomised controlled trial**	Shoulder	0.3×25mm,	Aμ350	90 seconds
**Moreno 2015** ^[17]^ **Randomised controlled trial**	Shoulder	On depth estimation, specifics not given	6mA	3 doses of 4 seconds
**Abat et al., 2016** ^[7]^ **Randomised controlled trial**	Knee	Not documented	2mA at three locations in tendon	Until area debrided
**Ronzio et al., 2017** ^[8]^ **Randomised controlled trial**	Ankle	0.22×13mm	100μA -450μA current density of 5.86mA/cm^2^	3 doses per session 20 seconds EPI, rest 40 seconds (total 3min)
**Moreno et al., 2017** ^[15]^ **Randomised controlled trial**	Thigh	0.33×50mm	3mA	3 doses of 5 seconds each
**Valera-Garrido et al., 2010** ^[18]^ **Uncontrolled study**	Knee	Not documented	4–6mA	3 doses of 3 seconds
**Valera-Garrido et al., 2014** ^[9]^ **Uncontrolled study**	Elbow	0.3×25mm	4–6mA	Approximately 3 doses of 3 seconds
**Abat et al., 2014** ^[19]^ **Uncontrolled study**	Knee	0.3×0.32mm	3mA	Until debrided
**Abat et al., 2015** ^[6]^ **Uncontrolled study**	Knee	0.3×length required	3mA	Until debrided
**Munoz et al., 2012** ^[20]^ **Uncontolled/Cost-effective study**	Elbow	Not documented	4–6mA	3 seconds

μA, microampere; mA, milliampere

**Table 6:** Results of Cochrane risk-of-bias tool for quality assessment for randomised controlled trials

Study and level of evidence	Random sequence generation	Allocation concealment	Blinding of participants and personnel	Blinding of outcome assessment	Incomplete outcome data	Selective reporting	Other bias
**Arias-Buria et al., 2015**^[10]^ **1b**	+	+	?	?	+	?	?
**Valtiera et al., 2018**^[16]^ **1b**	+	+	+	+	+	?	?
**Moreno MD, 2015**^[17]^ **2b**	−	+	?	−	−	−	−
**Abat et al., 2016**^[7]^ **1b**	+	+	+	−	+	?	−
**Ronzio et al., 2017**^[8]^ **2b**	−	?	−	?	−	−	−
**Moreno et al., 2017**^[15]^ **2b**	+	+	+	−	−	−	−

+ indicates low risk of bias; − indicates high risk of bias; ? indicates medium risk of bias.
